# Thermal Optimization of Edge-Emitting Lasers Arrays

**DOI:** 10.3390/ma18010107

**Published:** 2024-12-30

**Authors:** Robert P. Sarzała, Dominika Dąbrówka, Maciej Dems

**Affiliations:** Institute of Physics, Lodz University of Technology, ul. Wólczańska 217/221, 90-003 Łódź, Poland; dominika.dabrowka@dokt.p.lodz.pl

**Keywords:** nitride edge-emitting laser arrays, thermal optimization, computer simulation, GaN

## Abstract

This paper presents a novel approach to address the issue of uneven temperature distribution in one-dimensional laser arrays, specifically in gallium nitride edge-emitting lasers emitting green light of 540 nm. The results were obtained using heat flow numerical analysis, which included an optimization method specifically developed for this type of array. It was demonstrated that thermal optimization of a one-dimensional edge-emitting laser array can be achieved by adjusting the placement of the emitters within the array and the size of the top gold contact, without changing the overall dimensions of the device. The proposed design alterations ensure an even temperature distribution across the array without the need for a complex and expensive cooling systems. The proposed optimization method can be applied to arrays made from various material systems, including nitrides, arsenides, and phosphides.

## 1. Introduction

In recent years, nitride-based semiconductor lasers emitting green light have garnered significant interest due to their potential applications in various fields. These lasers are utilized in laser displays, lighting systems, projection systems, and fiber-optic telecommunications. There is also a demand for compact green lasers suitable for small (micro- or pico-scale) portable projectors, semiconductor lighting, medical equipment, and military applications, as the green light beam exhibits weak dependence on external conditions [1,2,3,4,5,6]. The development of arrays based on these lasers would enable achieving higher emitted powers than those provided by single emitters, significantly expanding the range of applications for these devices. However, currently, there are no commercially available green light sources (>500 nm), let alone their arrays [1,2,3]. For laser emitters based on nitride materials, commercial production has so far only mastered edge-emitting lasers (EELs) and their one-dimensional arrays emitting wavelengths from violet to blue–green light [7,8,9,10,11,12].

Laser arrays, or semiconductor laser systems, are key components of many modern technologies. They consist of multiple individual emitters placed in close proximity in an orderly configuration. Despite their widespread use across various fields, several challenges can limit their performance and application. One of the main challenges with laser arrays is effective thermal management [7,8,9,13]. The simultaneous operation of multiple emitters generates significant heat, which can lead to excessive heating of the device. This, in turn, may alter the operating parameters of the array, reduce its efficiency, and shorten its lifespan. In particular, an increase in temperature degrades material properties, such as reducing thermal and electrical conductivity. Moreover, temperature rise alters the band gap of the materials forming the active region of the laser, leading to a shift in the gain spectrum of the active region and a reduction in its value, negatively affecting the emitted power. The significant amounts of heat generated also contribute to uneven temperature distribution across the individual emitters in the array, particularly within their active regions. This leads to uneven operation of the emitters, which not only switch on and off asynchronously but also emit light at different wavelengths, thereby degrading the output beam quality of the entire array [14]. These issues are closely related to the effect of emitter mutual heating (thermal crosstalk), which occurs primarily within the array itself—rather than at the solder/heatsink interface or within the housing—critically impacting the performance of laser bars [15]. Therefore, precise management of heat flow between individual emitters in laser arrays is crucial for further improving their efficiency.

The thermal crosstalk effect in emitter arrays has been analyzed since the 1970s [16,17,18]. Scientists have analyzed it in one-dimensional Distributed Feedback Lasers arrays [19], arrays made of arsenide materials [15,20,21,22], and in arrays made of nitride materials [23,24,25]. They mainly focused on investigating the impact of the emitter spacing [19,26], the substrate dimensions [22,25,26], the material of the heat sink [19], and the dimensions of selected layers forming a single emitter or the entire array [20,22,25]. Other analyzed factors include the impact of additional heat spreaders, such as diamond-based ones [24], on temperature rise within the array.

Temperature equalization in the active regions of individual emitters of the array and maintaining it at the lowest possible level are crucial for improving its efficiency and longevity. Therefore, scientists are intensively working on developing appropriate methods and solutions, as well as optimizing the array design. One of the simplest solutions is to significantly increase the distance between individual emitters. Unfortunately, this results in a loss of the array’s compactness. Specialized systems are also used to individually control the power supply of each emitter, but this solution complicates the device’s construction and increases its cost. Considerable attention has been devoted to improving commonly used solutions for temperature equalization and reduction, such as stackable micro-channel coolers and impingement-type heat exchangers. Over the years, a number of innovations have been developed that have improved cooling efficiency [27,28,29,30,31,32,33,34]. Studies have also been conducted on the impact of solder materials on the thermal performance and reliability of arrays [35,36,37,38]. Another approach to addressing the thermal issues of the array was presented in a 2018 paper, which analyzed the thermal properties of mini-laser arrays based on GaN, consisting of five laser bars in a TO-9 package [26]. It was observed that the temperature difference between evenly spaced bars exceeded 5 K, but by modifying the width of the bars and the spacing between them, this difference was reduced to less than 0.4 K. The outer bars were widened, while the remaining three were narrowed, while maintaining the same total width of the bars. Increasing the volume of the AlN substrate and changing its shape to a trapezoidal form further optimized the temperature distribution. In the present work, we propose a much simpler solution to this problem. The control of self-heating in the emitters can be achieved by properly selecting the arrangement of the individual emitters in combination with the appropriate width of the gold contact on the top of the device, as will be demonstrated in this paper. This approach eliminates the need for changes in bar widths, which could potentially affect optical modes.

The proposed solution is demonstrated through a case study of a multi-element laser bar made from edge-emitting lasers that emit green light at a wavelength of 540 nm. The main goal of the work was to search for design solutions that could equalize and reduce the temperature in the aforementioned array while maintaining its width. The solutions were sought by utilizing the self-heating effect of the emitters in the array. It was noticed that this generally unfavorable phenomenon could, under certain conditions, contribute to the equalization of temperatures in all emitters in the array. By appropriately adjusting the distance between selected emitters, it is possible to raise the temperature in some emitters while lowering it in others, effectively reducing the temperature differences. However, it turned out that simply adjusting the emitter positions is insufficient for full temperature equalization. Therefore, an additional solution was sought by modifying the width of the upper gold contact, which allowed us to adjust the temperature of the outermost emitters. The proposed approach ensures that all emitters in the array, especially their active regions, maintain the same temperature.

Although this work is purely theoretical and numerical, it focuses on the practical implementation of efficient high-power laser arrays. All known technological limitations of gallium nitride light emitters have been carefully considered. Therefore, the results obtained in this study can serve as a foundation for the design of new devices. The proposed solutions can be extended to arrays made from various material systems, including arsenides, phosphides, and antimonides.

## 2. Materials and Methods

### 2.1. Structure

**Figure 1:**
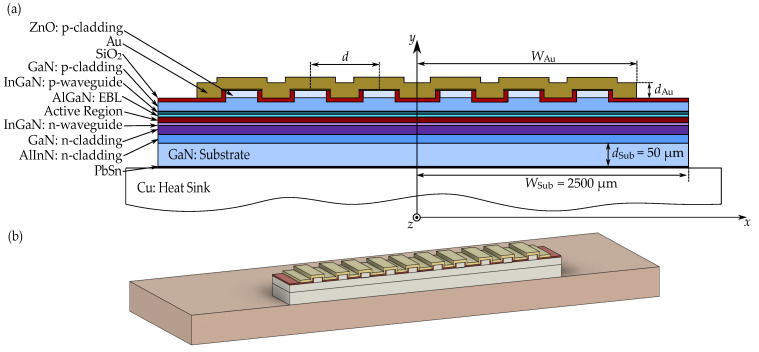
Schematic (**a**) of the laser bar based on edge-emitting nitride lasers, with marked layers that make up the structure, as well as key parameters: $d_{Au}$—thickness of the top gold contact, $W_{Au}$—width of the top gold contact, $d_{Sub}$—thickness of the substrate, $W_{Sub}$—width of the substrate. Three-dimensional visualization (**b**) of a 10-element laser bar. The drawings are not to scale.

The thermal optimization was conducted for edge-emitting lasers with a ridge waveguide. Each emitter was designed to emit green light at 540 nm [39]. The structure is based on a gallium nitride (GaN) substrate, 2500 µm in width and 50 µm in thickness. Directly above it is an n-type layer of ${\mathrm{Al}}_{0.83}{\mathrm{In}}_{0.17}$N, composition-matched to the underlying GaN. These two layers function as the n-type cladding layers. Next is an n-type waveguide layer of ${\mathrm{In}}_{0.08}$${\mathrm{Ga}}_{0.92}$N. The active region consists of three 2.7 nm ${\mathrm{In}}_{0.29}$${\mathrm{Ga}}_{0.71}$N quantum wells, surrounded by four 10 nm GaN barriers. Directly above the active region is a p-${\mathrm{Al}}_{0.20}$${\mathrm{Ga}}_{0.80}$N electron blocking layer (EBL) and a p-type waveguide made of ${\mathrm{In}}_{0.08}$${\mathrm{Ga}}_{0.92}$N. The p-type cladding layer, consisting of a p-GaN layer and a ZnO layer, is located above the waveguide. The ZnO layer has already been used in lasers as an optical confinement layer [40,41,42]. A ridge waveguide with a width of 2 µm was fabricated in these layers. A gold contact is situated on top of the emitter, insulated from the structure by a silicon dioxide layer except at a small area atop the ridge waveguide. A schematic and a three-dimensional visualization of the edge-emitting laser array are shown in Figure 1, and detailed design data of the analyzed structure is provided in Table 1. In Figure 1a, the individual layers of the array and the most important dimensions are labeled. The width of the top gold contact is denoted as $W_{Au}$ and its thickness as $d_{Au}$.

The emitters forming the optimized array are primarily composed of materials from the III-N group, including binary GaN and ternary compounds such as AlGaN, InGaN, and AlInN. For these materials, a temperature-dependent thermal conductivity relationship has been applied, expressed using the temperature coefficient δ as follows:

(1) $$k\left(T\right)=k_{300K}{\left(\frac{T}{300\;K}\right)}^{\delta },$$

where $k_{300K}$ is the thermal conductivity at room temperature [W/(m·K)], and *T* is the temperature [K]. The relationship given in Equation (1) is commonly found in the literature and is applied to nitride materials. For the mentioned layers, the temperature coefficient δ is −1.4 [23,43].

The thermal conductivities at room temperature for the binary material GaN were taken from [43,44]. For the ternary materials, i.e., ${\mathrm{Al}}_{x}$${\mathrm{Ga}}_{1-x}$N and ${\mathrm{In}}_{y}$${\mathrm{Ga}}_{1-y}$N, the thermal conductivities were calculated based on the known values of thermal conductivity at room temperature for binary materials using linear interpolation with a curvature coefficient *c*, which indicates the deviation from linearity [23]. For calculating the thermal conductivity of ${\mathrm{Al}}_{x}$${\mathrm{Ga}}_{1-x}$N, AlN and GaN were used [23,45], and for ${\mathrm{In}}_{y}$${\mathrm{Ga}}_{1-y}$N, InN and GaN were used [23,46]. For the AlInN material, the data from [47] were used as a reference.

Since the active region consists of seven layers with small thicknesses, an equivalent thermal conductivity was calculated for it. This structural simplification does not affect the accuracy of the results but significantly reduces memory requirements and computation time, which is particularly important for calculations involving laser arrays. A standard approach was used, in which the equivalent thermal conductivities in the parallel direction $k_{x}$ (along the *x*-axis) and in the perpendicular direction $k_{y}$ (along the *y*-axis) to the layer were calculated for the layered materials. These conductivities were calculated using the following formulas [48]:

(2) $$\begin{array}{cc} k_{x} & =\frac{\sum_{i}^{N}d_{i}k_{i}}{d_{tot}}, \end{array}$$

(3) $$\begin{array}{cc} k_{y} & =\frac{d_{tot}}{\sum_{i}^{N}\frac{d_{i}}{k_{i}}}, \end{array}$$

where *N* is the number of combined layers, $d_{i}$ is the thickness of the *i*-th combined layer, $k_{i}$ is the thermal conductivity of the *i*-th combined layer, and $d_{tot}$ is the total thickness of the combined layers. For the combined layers, the base thermal conductivities are 2.00 W/(m·K) for the quantum wells made of n-${\mathrm{In}}_{0.29}$${\mathrm{Ga}}_{0.71}$N and 61.00 W/(m·K) for the GaN barriers. A temperature-dependent thermal conductivity relationship, as described by Equation (1), was also applied for the active region.

The simulated device comprises not only semiconductor materials of the III–N group but also other materials such as gold (Au), silicon dioxide (${\mathrm{SiO}}_{2}$), zinc oxide (ZnO), copper (Cu), and a tin–lead alloy (PbSn), which serves as a thermal contact layer between the copper heatsink and the GaN substrate. In the calculations, temperature-dependent effects were not considered for some materials, as the temperature variations within the analyzed range were small and did not significantly affect the final results. The thermal conductivity data were taken from [49,50] for Au, ${\mathrm{SiO}}_{2}$, PbSn, and Cu. For ZnO material, significant discrepancies exist in the literature regarding the reported thermal conductivity values [51]. Furthermore, its heat management can be significantly impacted by the morphology of the ZnO layer. The value used in the calculations was selected based on studies describing the application of a ZnO layer as a cladding layer [40,41,42].

Table 1 presents the thermal conductivities of the materials forming the analyzed structure, determined for three temperatures: 300 K, 400 K, and 500 K.

It must be noted that the design of the nitride laser diode emitting green light that we used is just one example. It shows very interesting properties, which is why we chose it. Nevertheless, the proposed optimization method can be applied to other types of laser arrays, including those emitting light of different wavelengths, made from various materials, such as arsenides, phosphides, or antimonides.

### 2.2. Numerical Model

The heat flow and temperature distribution in the analyzed structures were modeled using the Finite Element Method (FEM) to solve the heat flow equation:

(4) $$\nabla \;\cdot \;\left(\lambda_{T}\;\nabla T\right)=-g,$$

where *T* is the temperature, $\lambda_{T}$ is the thermal conductivity, and *g* is the heat source density. It is assumed that all the heat is generated in the active region. While this is not entirely accurate, carrier recombination in the active region contributes to the vast majority of heating in correctly manufactured devices. Additionally, other heat sources, such as contacts and high-resistive layers, are located in close proximity to the active region (relative to the overall array dimensions), and their impact can be considered within the active region heat source density. The device temperature at the bottom of the 5 mm thick heatsink is assumed to be maintained at 300 K.

Calculations were performed using the multi-physics modeling software PLaSK, developed by the authors [52] and available as open source [53]. It is a comprehensive tool for the numerical analysis of a broad range of physical phenomena in photonic devices. Although it is primarily designed for simulating semiconductor lasers, its range of applications includes transistors, light-emitting diodes, photodetectors, and more. Due to its modular nature, it is possible to perform computations of virtually any physical phenomenon in micro-scale structures.

Since its advent, PLaSK has been used in numerous scientific publications. Investigated phenomena included thermal, electrical, and optical analysis in edge-emitting lasers [39,54], advanced vertical-cavity surface-emitting lasers [55,56], photonic-crystal lasers [57], photodetectors [58], and high-contrast grating lasers [59]. Simulation results obtained with it have shown very good agreement with experimental data [59,60,61,62], proving the reliability and accuracy of the software.

In this work, we utilized the PLaSK module for the analysis of heat dissipation using the Finite Element Method. It employed rectangular bilinear elements, with a total mesh size of approximately 100,000 elements per emitter. Due to the mirror symmetry of the entire array, only half of it was modeled, with adiabatic boundary conditions applied along the central plane. However, due to the temperature dependence of the thermal conductivity throughout the structure, an iterative approach was necessary to solve the heat flow equation. Consequently, the total computation time varied from seconds for small arrays (fewer than 10 emitters) to 20–30 min for larger arrays comprising around 20 emitters.

PLaSK by default implements such an iterative process and considers the temperature dependence of the material parameters within its default material database, which is compiled from multiple available sources. Additionally, it allows for refinement of these parameters for the particular structure under investigation. For the analysis presented in this article, we used the relations shown in Table 1 and Equation (1).

The optimization procedure was performed with the SciPy [63] package. The method used was Sequential Least Squares Programming (SLSQP) [64], with constraints ensuring that no emitters overlap. The figure of merit was the standard deviation of the temperature in the active regions of all emitters. To prevent undesired solutions where emitters combine in pairs (each pair comprising two emitters very close to each other and mutually heating), which significantly increased both the maximum and average temperature of the entire array, an additional constraint was introduced. We required that the distances between consecutive emitters increase progressively towards the center of the array. Thus, the emitters near the array edges were closest to each other, while those in the center were the most distant. This approach not only reduced the temperature difference between all the array emitters but also slightly decreased the maximum temperature of the entire device.

## 3. Results and Discussion

**Figure 2:**
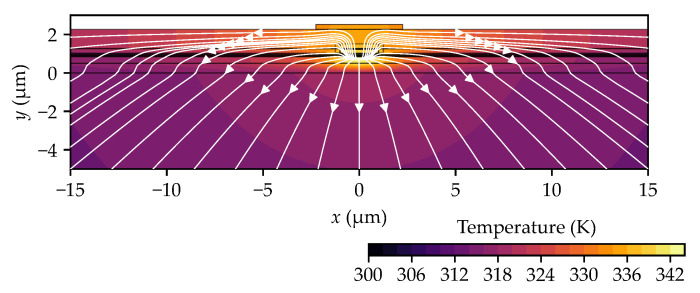
Example of the temperature distribution with directions of heat flow generated in the active region marked by white arrows.

As mentioned earlier, in edge-emitting lasers, heat is primarily generated in the active region, where laser radiation is emitted. Consequently, this region experiences the highest temperature increase, with the generated heat spreading outward to external components, primarily towards the heat sink. In our study [25], we demonstrated that if the thickness of the top metallization layer (mainly composed of gold) exceeds 1 µm, this contact can function as a heat spreader, significantly reducing the maximum temperature increase in the active region. The efficiency of heat dissipation through the top contact can be enhanced by introducing layers with the highest possible thermal conductivity between the active region and the contact. For the laser structure analyzed in this study, heat dissipation efficiency can be improved by replacing the cladding layer material, made of ITO, with ZnO. The use of a ZnO layer facilitates effective heat transfer to the gold contact, spreading the heat over a wider area of the structure. This, in turn, promotes more efficient heat removal from the laser’s interior. An example of heat distribution in an edge-emitting laser is shown in Figure 2.

**Figure 3:**
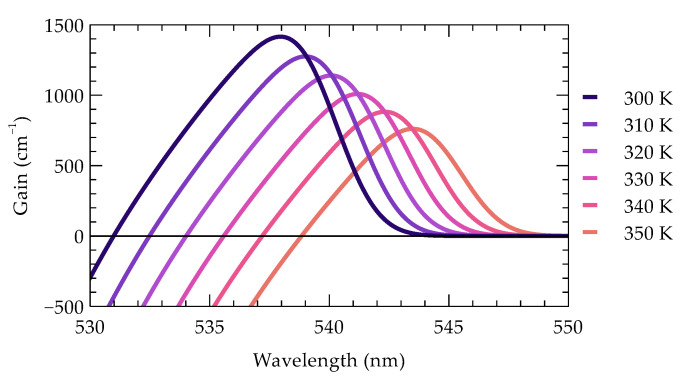
The effect of temperature on the gain spectrum of the active region in a nitride-based edge-emitting green laser.

Nitride materials, such as GaN, InGaN, InAlN, and AlN, exhibit significant shifts in emission wavelength as a function of temperature, which impact the stability and performance of optoelectronic devices like LEDs and edge-emitting lasers. The dλ/dT values for these materials typically range from 0.1 nm/K to 0.4 nm/K, depending on the device structure and operating conditions [65,66,67,68,69]. For the structure under consideration, this is illustrated in Figure 3, which shows the calculated material gain in the laser’s active region as a function of wavelength for various temperatures. As the temperature increases, a reduction in gain is observed, accompanied by a red shift in the gain peak relative to the target emission wavelength. Consequently, temperature affects other parameters, such as threshold current and efficiency, emphasizing the importance of effective thermal management in these devices, especially under extreme conditions, where wavelength shifts can reach up to 0.5 nm/K.

For a 20-element non-optimized array operating at 1 W per emitter, the spectral non-uniformity is 10.4 nm, assuming a wavelength shift of 0.4 nm/K resulting from a maximum temperature difference of 26 K between the emitters. However, with optimization, the spectral non-uniformity is significantly reduced to an extremely low value of 0.02 nm.

**Figure 4:**
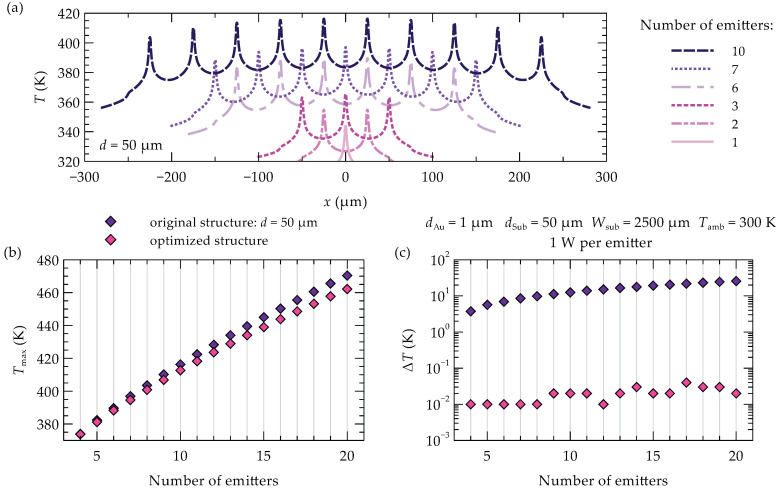
Temperature profiles (**a**) in the active region depending on the number of evenly spaced emitters in a one-dimensional array. Maximum temperature (**b**) in the laser bar and maximum temperature differences (**c**) between emitters in the array as a function of the number of emitters. In graphs (**b**,**c**), results for arrays with evenly spaced emitters are shown in purple, while results for laser bars with optimally adjusted distances between emitters and tailored gold width are shown in pink.

In laser arrays, temperatures are even higher than in single emitters. This is primarily due to the previously mentioned thermal crosstalk effect, which involves mutual heating among the emitters within the array. The self-heating of individual emitters in the array intensifies as the number of emitters in the laser bar increases and/or the distance between them decreases. With an increasing number of emitters, not only does the maximum temperature in the array rise (see Figure 4a,b), but greater temperature unevenness is also observed among the individual emitters in the array (see Figure 4b,c). The differences in maximum temperature increases between emitters, particularly between the central and edge emitters in the array, can reach several tens of percent. For the analyzed 10-element laser bar, a temperature difference of 12.5 K (11%) is observed between the central and edge emitters; doubling the number of emitters in the array doubles this temperature difference. Such significant temperature variations between emitters lead to differences in material gain and emitted wavelengths among the lasers, resulting in uneven operation across the array.

**Figure 5:**
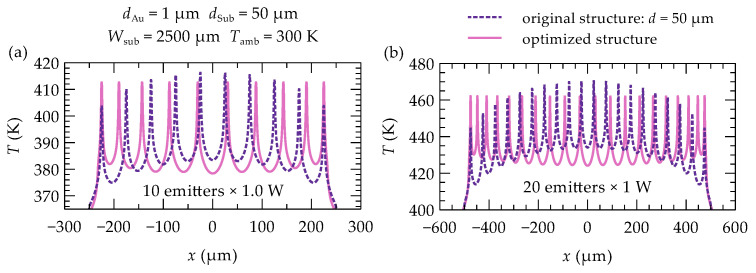
Temperature profile in the active region of the laser bar with (**a**) 10 emitters and (**b**) 20 emitters, each with a heat source power of 1 W, before (dashed line) and after (solid line) optimization.

**Figure 6:**
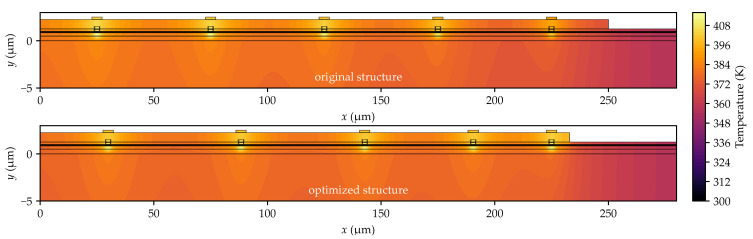
Temperature distribution in a 10-element one-dimensional laser bar before and after optimization. Diagram not to scale. Because of the mirror symmetry of the structure, only half of it is shown.

The thermal crosstalk phenomenon, although generally detrimental to heat dissipation, can be utilized to equalize the maximum temperatures across individual emitters in a laser array. The proposed solution involves unevenly spacing the emitters within the array. This approach imposes a constraint: the positions of the outermost emitters remain fixed to ensure that the overall device size is identical to that of an array with evenly spaced emitters. Furthermore, selecting an appropriate width ($W_{Au}$) for the gold layer on top of the array (see Figure 1a) is crucial. This layer serves as the p-side electrical contact and acts as a heat spreader, facilitating lateral heat distribution within the laser bar. The developed optimization method enables the determination of emitter spacing and gold contact width that result in nearly identical temperatures in the active regions of the array emitters (see Figure 4c and Figure 5). In optimized multi-emitter arrays, these temperature differences do not exceed 0.02 K, and in larger arrays, they are no more than 0.05 K. The effect of these modifications on the temperature profiles in the emitters’ active regions is shown in Figure 5, which compares the temperature profiles before (dashed line) and after (solid line) optimization for two arrays: (a) a 10-emitter array and (b) a 20-emitter array. Additionally, Figure 6 illustrates the temperature distribution in a 10-emitter laser bar before and after optimization.

Temperature equalization across all emitters is possible because moving the outer emitters closer raises their temperatures, while increasing the spacing between central emitters improves heat dissipation from the center of the array. This approach lowers the temperature in the central part and raises it on the sides of the array, resulting in a more uniform temperature distribution. The temperature increase in the outer emitters is also influenced by a 17 µm reduction in the width of the gold contact layer (compared to the array with evenly spaced emitters—see Figure 6). This narrowing limits the surface area over which heat spreads and restricts its flow to the heat sink, thereby increasing the temperature of the outer emitters.

Proper selection of the gold contact layer width allows not only for temperature equalization across the outermost emitters but also for a reduction in the maximum temperature across the entire array (see Figure 4b and Figure 5). This effect is particularly pronounced in arrays with a larger number of emitters. For instance, in a 20-emitter array, the maximum temperature is reduced by 2% compared to the unoptimized structure, while in a smaller array (e.g., 10 emitters), the reduction is approximately 1%.

**Figure 7:**
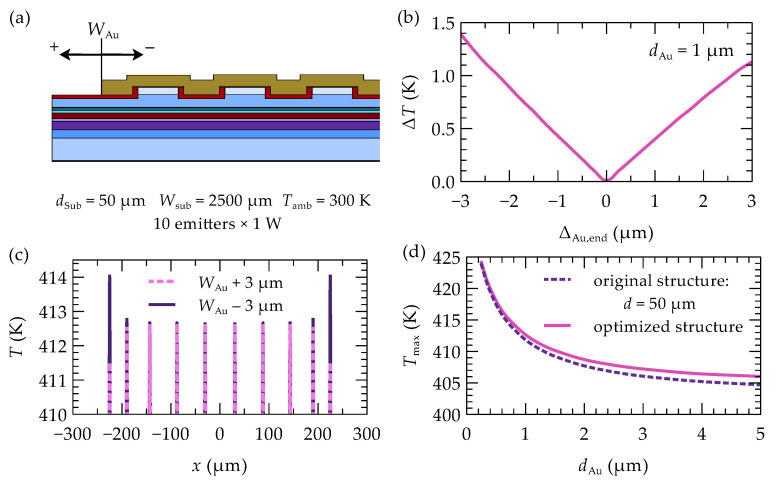
Impact of manufacturing precision of the gold width: (**a**) schematic of the examined precision, (**b**) maximum temperature difference between the emitters of the optimized 10-element array as a function of the dimensional deviation from the recommended gold width, (**c**) temperature profile section of the optimized array with gold layers 3 µm longer and shorter, and (**d**) impact of the gold contact thickness on the maximum temperature in the array.

The recommended dimensions of the gold layer, determined through simulations, are specified with a precision of up to thousandths of a micrometer. However, considering technological capabilities, the impact of manufacturing precision for this layer on the effectiveness of the proposed optimization was investigated. A manufacturing error range of ±3 µm for the gold layer was considered (see Figure 7a). The results, shown as a graph of the maximum temperature difference (ΔT) between the emitters in the array as a function of the gold layer’s manufacturing precision, are presented in Figure 7b. Making the gold contact wider than the dimensions obtained during optimization reduces the temperature of the outer emitters due to better heat dissipation from the structure. However, this also increases the temperature unevenness between emitters. In the case of a gold layer that is 3 µm wider in the analyzed 10-emitter array, the temperature difference between the central and outermost emitters is 1.1 K. On the other hand, if the gold layer is made 3 µm narrower, the temperature difference increases to 1.3 K. A smaller amount of gold limits and hinders the heat spread from the outer emitters, thereby raising their temperature. This effect is most noticeable in the two outer emitters, but the manufacturing precision of the gold layer does not significantly impact the maximum temperatures in the central emitters, as shown in Figure 7c. Compared to the temperature differences between emitters before optimization (12.5 K), the changes within the considered range are relatively small and are unlikely to have a significant impact.

As mentioned earlier, the thickness of the gold contact can significantly affect the temperature within the device. A thicker gold layer causes the heat to spread more widely, allowing it to flow more efficiently toward the heat sink at the bottom of the array. According to Fourier’s law, a wider heat flux (i.e., lower flux density) at the same vertical distance to the heat sink results in a smaller temperature gradient within the structure. As a result, the temperature inside the array decreases, as shown in Figure 7d. However, beyond a certain thickness of the gold layer, no further significant temperature reductions are observed.

**Figure 8:**
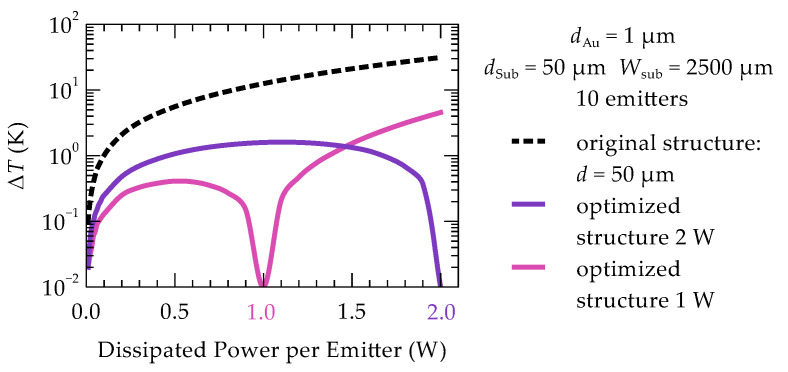
Temperature difference between the central and outermost emitter in the 10-element optimized array as a function of the dissipated power in each heat source: while maintaining the emitter arrangement obtained during the optimization of the 10-emitter array with 1 W power per emitter (pink line), for the optimal arrangements obtained for 2 W power (purple line), and the difference in the unoptimized structure (dashed line).

The presented optimization results were obtained under the assumption that each emitter dissipates 1 W of power. However, varying this value does not lead to significant changes. In other words, the optimized structure shows robustness for a large range of powering. The impact of the dissipated heat in individual emitters of the array on the maximum temperature differences (ΔT) between the emitters is shown in Figure 8. Reducing the dissipated power in the laser leads to slight temperature changes in the emitters, but the temperature unevenness does not exceed 0.4 K. On the other hand, at higher powers, a faster increase in temperature unevenness between the emitters is observed. Increasing the power from 1.0 W to 1.5 W results in a maximum temperature difference of 1.5 K (purple line in Figure 8). Therefore, it is most appropriate to perform optimization for the maximum assumed power at which the emitters will operate. An example of performing optimization for higher power and applying it to lower powers is shown in Figure 8 (pink line). With this approach, the maximum temperature difference between emitters increases, but this increase remains relatively small and does not exceed 1.6 K for all lower power values compared to the power for which the optimization was performed.

## 4. Conclusions

This paper presents an innovative method for equalizing the temperatures of individual emitters in an array, demonstrated using a one-dimensional array of edge-emitting lasers emitting green light. Temperature equalization was achieved by optimizing the arrangement of the inner emitters and adjusting the width of the array’s upper gold contact, without altering the position of the outermost emitters. The proposed solution enables uniform temperature distribution in each emitter of the array without the need for specialized power supply or cooling systems. The introduced modifications not only equalize the temperature but also contribute to a slight reduction in the overall temperature. For a 10-emitter array, the maximum temperature increase is 116.30 K, with a temperature increase difference between emitters of 11% relative to the maximum increase. Using the proposed modifications, the overall temperature increase was reduced by 3%, and the difference in temperature increase between emitters was minimized to nearly 0% of the maximum increase before optimization (0.01 K). The proposed method is effective for both small arrays and larger arrays, such as those with 20 emitters. In the case of a 20-emitter array, the temperature increase is reduced by nearly 5%. Prior to optimization, the temperature increase difference between emitters reached 15% of the maximum increase, but after optimization, this difference was reduced to nearly 0% (0.02 K).

Determining the appropriate geometric parameters is made possible through a developed and customized computational optimization method tailored to the analyzed array. The phenomenon of thermal crosstalk, which typically causes unfavorable thermal effects, as demonstrated by the presented results, can also be harnessed to improve the operating conditions of the laser array, thereby enhancing its performance parameters. Furthermore, improving the thermal conditions of arrays solely through structural modifications can be crucial for achieving low-cost, high-power devices. It is worth noting that the proposed optimization method is applicable to dies made from any material system, such as nitrides, arsenides, or phosphides.

## Figures and Tables

**Table 1:** Design data of the modeled edge-emitting lasers and thermal parameters of materials used in simulations: $k_{300\;K}$, $k_{400\;K}$, and $k_{500\;K}$ are thermal conductivities at 300 K, 400 K, and 500 K, respectively. QW stands for quantum well and EBL for electron blocking layer.

Layer		Material	Thickness (µm)	Doping (${\mathrm{cm}}^{-3}$)	$k_{300K}$ (W/(m·K))		$k_{400K}$ (W/(m·K))		$k_{500K}$ (W/(m·K))	
p-side contact		Au	1.00	-	317.10		310.70		304.30	
Insulator		${\mathrm{SiO}}_{2}$	0.25	-	1.29		1.45		1.56	
p-cladding		ZnO	0.28	-	50.00		50.00		50.00	
		p-GaN	0.28	Mg: $2\times 10^{19}$	92.00		61.50		45.00	
p-waveguide		p-${\mathrm{In}}_{0.08}$${\mathrm{Ga}}_{0.92}$N	0.045	Mg: $1\times 10^{19}$	23.00		15.37		11.25	
EBL		p-${\mathrm{Al}}_{0.20}$${\mathrm{Ga}}_{0.80}$N	0.01	Mg: $5\times 10^{19}$	13.00		8.69		6.36	
Active region	3 × QW 4 × barrier	${\mathrm{In}}_{0.29}$${\mathrm{Ga}}_{0.71}$N GaN	0.0027 0.01	Si-doped -	$k_{x}$ 48.46	$k_{y}$ 8.39	$k_{x}$ 32.39	$k_{y}$ 5.61	$k_{x}$ 23.70	$k_{y}$ 4.10
n-waveguide		n-${\mathrm{In}}_{0.08}$${\mathrm{Ga}}_{0.92}$N	0.01	Si: $5\times 10^{18}$	28.00		18.72		13.70	
n-cladding		n-GaN	0.35	Si: $2\times 10^{18}$	50.00		50.00		50.00	
		n-${\mathrm{Al}}_{0.83}$${\mathrm{In}}_{0.17}$N	0.50	Si: $5\times 10^{18}$	94.87		3.26		2.38	
Substrate		n-GaN	50.00	Si: $2\times 10^{18}$	166.00		110.97		81.19	
Solder		PbSn	1.00	-	50.00		50.00		50.00	
Heat sink		Cu	5000	-	400.80		392.47		386.13	

## Data Availability

The original contributions presented in this study are included in the article. Further inquiries can be directed to the corresponding authors.
